# Macrophage 11β-HSD-1 deficiency promotes inflammatory angiogenesis

**DOI:** 10.1530/JOE-17-0223

**Published:** 2017-07-04

**Authors:** Zhenguang Zhang, Agnes E Coutinho, Tak Yung Man, Tiina M J Kipari, Patrick W F Hadoke, Donald M Salter, Jonathan R Seckl, Karen E Chapman

**Affiliations:** 1 University/BHF Centre for Cardiovascular ScienceThe Queen’s Medical Research Institute, The University of Edinburgh, Edinburgh, UK; 2 Centre for Genomic and Experimental MedicineMRC Institute of Genetic and Molecular Medicine, The University of Edinburgh, Western General Hospital, Edinburgh, UK

**Keywords:** steroid metabolism, glucocorticoid, inflammation, 11β-HSD1, macrophage

## Abstract

11β-Hydroxysteroid dehydrogenase-1 (11β-HSD1) predominantly converts inert glucocorticoids into active forms, thereby contributing to intracellular glucocorticoid levels. 11β-HSD1 is dynamically regulated during inflammation, including in macrophages where it regulates phagocytic capacity. The resolution of inflammation in some disease models including inflammatory arthritis is impaired by 11β-HSD1 deficiency or inhibition. However, 11β-HSD1 deficiency/inhibition also promotes angiogenesis, which is beneficial in mouse models of surgical wound healing, myocardial infarction or obesity. The cell types responsible for the anti-inflammatory and anti-angiogenic roles of 11β-HSD1 have not been characterised. Here, we generated *Hsd11b1^MKO^* mice with *LysM-Cre* mediated deletion of *Hsd11b1* to investigate whether 11β-HSD1 deficiency in myeloid phagocytes is pro-angiogenic and/or affects the resolution of inflammation. Resolution of inflammatory K/BxN-induced arthritis was impaired in *Hsd11b1^MKO^* mice to a similar extent as in mice globally deficient in 11β-HSD1. This was associated with >2-fold elevation in levels of the endothelial marker *Cdh5* mRNA, suggesting increased angiogenesis in joints of *Hsd11b1^MKO^* mice following arthritis. A pro-angiogenic phenotype was confirmed by measuring angiogenesis in subcutaneously implanted polyurethane sponges, in which *Hsd11b1^MKO^* mice showed 20% greater vessel density than their littermate controls, associated with higher expression of *Cdh5*. Thus, 11β-HSD1 deficiency in myeloid phagocytes promotes angiogenesis. Targeting 11β-HSD1 in macrophages may be beneficial in tissue repair.

## Introduction

Glucocorticoids exert anti-inflammatory effects both *in vivo* and *in vitro*. Whilst the potent effects of synthetic glucocorticoids have been widely exploited clinically to treat inflammatory disease, including rheumatoid arthritis, the role of endogenous corticosteroids in regulating inflammation is less well understood (Coutinho & Chapman 2011). Nevertheless, macrophages have emerged as a key target for the anti-inflammatory and immunomodulatory effects of endogenous glucocorticoids (Bhattacharyya *et al*. 2007, Tuckermann *et al*. 2007). Macrophages orchestrate much of the tissue remodelling that follows injury (Minutti *et al*. 2016) and play a key role in the angiogenesis that is important for tissue repair (Nucera *et al*. 2011). Macrophages are also important in the excessive or dysregulated angiogenesis that contributes to the pathogenesis of many chronic inflammatory diseases, a maladaptive response in rheumatoid arthritis (Jackson *et al*. 1997, Koch 1998).

Macrophages express 11β-hydroxysteroid dehydrogenase type 1 (11β-HSD1), a glucocorticoid metabolising enzyme that, in intact cells, converts the intrinsically inert cortisone and 11-dehydrocorticosterone into the active glucocorticoids, cortisol and corticosterone, respectively (Chapman *et al*. 2013*b*). 11β-HSD1 thus amplifies glucocorticoid action. 11β-HSD1 deficiency or inhibition alters inflammatory responses (Chapman *et al*. 2013*a*). 11β-HSD1-deficient mice show more severe acute inflammation in thioglycollate-induced sterile peritonitis (Gilmour *et al*. 2006), following lipopolysaccharide (Zhang & Daynes 2007), in carrageenan-induced pleurisy (Coutinho *et al*. 2012), in the K/BxN serum-induced model of inflammatory arthritis (Coutinho *et al*. 2012) and following coronary artery ligation in a model of myocardial infarction (McSweeney *et al*. 2010). Macrophages are implicated in the altered inflammatory responses of 11β-HSD1-deficient mice. *In vitro*, peritoneal or splenic macrophages from 11β-HSD1-deficient mice overproduce inflammatory cytokines following lipopolysaccharide stimulation (Zhang & Daynes 2007). During thioglycollate-induced peritonitis, 11β-HSD1-deficient mice show a delay in the acquisition of phagocytic capacity by macrophages, though the inflammation resolves at a similar time to that in control mice (Gilmour *et al*. 2006). Reduced cholesterol accumulation in macrophages plays a role in the atheroprotective and anti-inflammatory effects of global 11β-HSD1 deficiency or inhibition in atherosclerosis-prone *Apoe^−^^/^^−^* mice (Garcia *et al*. 2013, Kipari *et al*. 2013, Luo *et al*. 2013). Following myocardial infarction, macrophages accumulate around the infarct zone more rapidly in 11β-HSD1-deficient mice (McSweeney *et al*. 2010). They switch to a pro-reparative, pro-angiogenic ‘M2’ phenotype earlier than in control mice, resulting in greater angiogenesis in the healing infarct and better recovery of heart function post-myocardial infarction (Small *et al*. 2005, McSweeney *et al*. 2010, Michailidou *et al*. 2012). Global 11β-HSD1 deficiency is also pro-angiogenic in other contexts: in the sponge implantation assay (Small *et al*. 2005); in adipose tissue of obese mice where it is associated with reduced adipose tissue hypoxia and inflammation (Michailidou *et al*. 2012) and in cutaneous surgical wounds, where it is associated with improved wound repair (Small *et al*. 2005).

Here, we have generated mice deficient in 11β-HSD1 in myeloid phagocytes (*Hsd11b1^MKO^*) to investigate the role of 11β-HSD1 activity in macrophages. We have used models of inflammation associated with a strong angiogenic response – the K/BxN serum transfer model of inflammatory arthritis and the sponge implantation assay, in which the role of 11β-HSD1 activity in macrophages is examined.

## Materials and methods

### Animals

All experiments on animals were carried out in accordance with the UK Home Office Animals (Scientific Procedures) Act of 1986 and European Directive 2010/63/EU, following approval by the University of Edinburgh Animal Welfare and Ethical Review Body. Mice were housed in groups (2–5 per cage) under controlled conditions: 12 h light/darkness cycle at 21°C with free access to standard rodent chow and water. *Hsd11b1^f/f^* mice, with *LoxP* sites flanking exon 3 of the *Hsd11b1 gene*, were generated by TaconicArtemis (Cologne, Germany) onto a C57BL/6 background (Verma M, Kipari TMJ, Zhang Z, Man TY, Forster T, Homer NZM, Seckl JR, Holmes MC & Chapman KE, unpublished observations). *Hsd11b1^MKO^* mice with myeloid cell 11β-HSD1 deficiency were generated by crossing *LysM-Cre* (backcrossed to a C57BL/6 background (Cramer *et al*. 2003)) with *Hsd11b1^f/f^* mice. Experimental (*Hsd11b1^MKO^*) and littermate control (*Hsd11b1^f/f^*) mice were the offspring of male *Hsd11b1^MKO^* mice bred with female *Hsd11b1^f/f^* mice. *Hsd11b1^Del1/Del1^* mice (Verma M, Kipari TMJ, Zhang Z, Man TY, Forster T, Homer NZM, Seckl JR, Holmes MC & Chapman KE, unpublished observations) were generated from *Hsd11b1^f/f^* mice and are homozygous for a germline deletion of exon 3 of the *Hsd11b1* gene. All experiments used male mice aged between 8 and 16 weeks.

### Peritoneal myeloid cell isolation and 11β-HSD1 activity assay

Mice were killed by CO_2_ asphyxiation. Resident cells were harvested from the peritoneum by lavage with 5 mL ice-cold PBS as previously described (Gilmour *et al*. 2006). Inflammatory cells elicited to the peritoneum 24 h, 72 h or 96 h following intra-peritoneal injection of thioglycollate (0.2 mL, 10%) were collected by lavage with 5mL ice-cold PBS (Coutinho *et al*. 2016). At 24 h, cells comprise mainly neutrophils and monocytes, whereas the population is predominantly macrophages at 72 h and 96 h (Melnicoff *et al*. 1989, Zhang *et al*. 2008, Coutinho *et al*. 2016). Where appropriate, neutrophils were isolated with anti-Ly6G antibody (clone RB6-8C5, Thermo Fisher Scientific) coupled to anti-rat IgG micro-beads (Miltenyi Biotec Ltd., Bisley, Surrey, UK) by magnetic-activated cell sorting as described previously (Coutinho *et al*. 2016). Macrophages were purified by adherence to tissue culture plates using an established procedure that gives >90% purity (Zhang *et al*. 2008). 11β-HSD1 activity in cultured cells was measured by addition of 5 nM [^3^H]-11-dehydrocorticosterone to the medium, as described previously (Coutinho *et al*. 2016).

### K/BxN serum transfer-induced arthritis

K/BxN serum was generated in-house as described previously (Coutinho *et al*. 2012). To induce arthritis in mice, 100 μL serum was injected intraperitoneally. Inflammation was scored daily for 21 days by an investigator blind to genotype. Scoring was carried out by visual examination according to a clinical index in which each joint was ascribed a score of 0–3, as described previously (Coutinho *et al*. 2012). The combined scores of all 4 limbs were calculated for each mouse. For histology, joints were fixed in 10% formalin for 1 day and then decalcified in 10% EDTA in neutral buffered formalin and paraffin embedded. Joint sections (4 μm) were deparaffinised, hydrated and stained with haematoxylin and eosin for histopathological examination. The severity of pathological changes in the ankle joint was quantified on a scale of 0–3 for each mouse, based on a scoring system modified from a previous protocol (Ruiz-Heiland *et al*. 2012). Briefly, the main pathological feature, synovium thickening, is assessed and assigned a score of 0 if there is no change compared to untreated, 1 if there is mild proliferation, 2 if there is extended proliferation and 3 if there is severe proliferation with tenosynovitis. RNA was extracted from the hind ankle joints using Trizol (Thermo Fisher Scientific) following pulverisation using a mortar and pestle, under liquid nitrogen.

### Sponge implant assay of angiogenesis

Mice were anaesthetised with isoflurane, and a sterilized sponge cube measuring 1 × 1 ×1 cm (Caligen Foam, Accrington, Lancashire, UK) was implanted subcutaneously on each flank, as described previously (Small *et al*. 2005). After 21 days, mice were killed and sponges were removed. The sponge from the right flank was frozen at 80°C for later RNA extraction using Trizol (Thermo Fisher Scientific). The sponge from the left flank was fixed in 10% formalin and embedded in paraffin. Sections were stained with haematoxylin and eosin for angiogenesis scoring. Vessel density was determined by Chalkley counting, as described previously (Small *et al*. 2005) by an investigator blind to experimental group. Briefly, a 25-point Chalkley eyepiece graticule (Graticules Ltd, Edenbridge, Kent, UK) was used to count blood vessels at ×250 magnification. The graticule was placed so that the maximum number of graticule dots overlay with blood vessels. The average count values (3 random areas/section, 2 sections/sample) were recorded as vessel numbers.

### Immunohistochemistry

Paraffin-embedded sections (4 µm) were deparaffinised, blocked with 10% goat serum after antigen retrieval with citrate buffer (pH 6) and incubated overnight with primary antibodies against isolectin IB4 (Alexa Fluor 488-conjugated isolectin B4, 1:100; I21411, Thermo Fisher Scientific) and α-smooth muscle actin (anti-actin, α-smooth muscle-Cy3 antibody, mouse monoclonal clone 1A4; C6198-100UL, Sigma-Aldrich) to detect endothelial cells and perivascular mural cells, respectively.

### Western blotting

Peritoneal cells or purified Ly6G^+^ cells were homogenised in RIPA buffer (R0278, Sigma-Aldrich) supplemented with proteinase inhibitor cocktail (1:100 dilution; P8340, Sigma-Aldrich) and phosphatase inhibitor (1:100 dilution; P2850, Sigma-Aldrich). Proteins (20 µg/sample) were separated by electrophoresis on a 4–12% Bis-Tris gel (NP0323BOX, Thermo Fisher Scientific) and then transferred to a 0.4 µm nitrocellulose membrane. Antibodies used for Western blotting recognise 11β-HSD1 (raised in sheep and kindly provided by Dr Scott Webster, the University of Edinburgh (De Sousa Peixoto *et al*. 2008, Coutinho *et al*. 2016)), β-tubulin (MAB3408, Merck Millipore) and GAPDH (ab9485, Abcam).

### RNA analysis

RNA (1 µg) was reverse transcribed into cDNA using a SuperScript III Reverse transcriptase system kit (Thermo Fisher Scientific). Levels of specific cDNAs were measured by quantitative (q)PCR in triplicate, using a LightCycler 480 (Roche) and Universal Probe Library (UPL; Roche)-based assays. Primer and probe information is shown in Supplementary Table 1 (see section on supplementary data given at the end of this article). A standard curve was prepared from pooled cDNA samples. Relative quantification was provided by LightCycler software using the maximum second derivative method and mRNA levels were normalised to an internal standard (chosen according to invariance between groups and validated against 18S RNA); *Tbp or Hprt*, as indicated in figure legends.

### Statistics

Values are means ± standard error of means (s.e.m.). Data were analysed using GraphPad Prism 5.0. Unpaired *t*-tests (with Welch’s corrections when variance was unequal), and one-way and two-way analysis of variance (ANOVA) were used, as appropriate. Significance was set at *P* < 0.05.

## Results

### Disruption of 11β-HSD1 in resident macrophages

**Figure 1:**
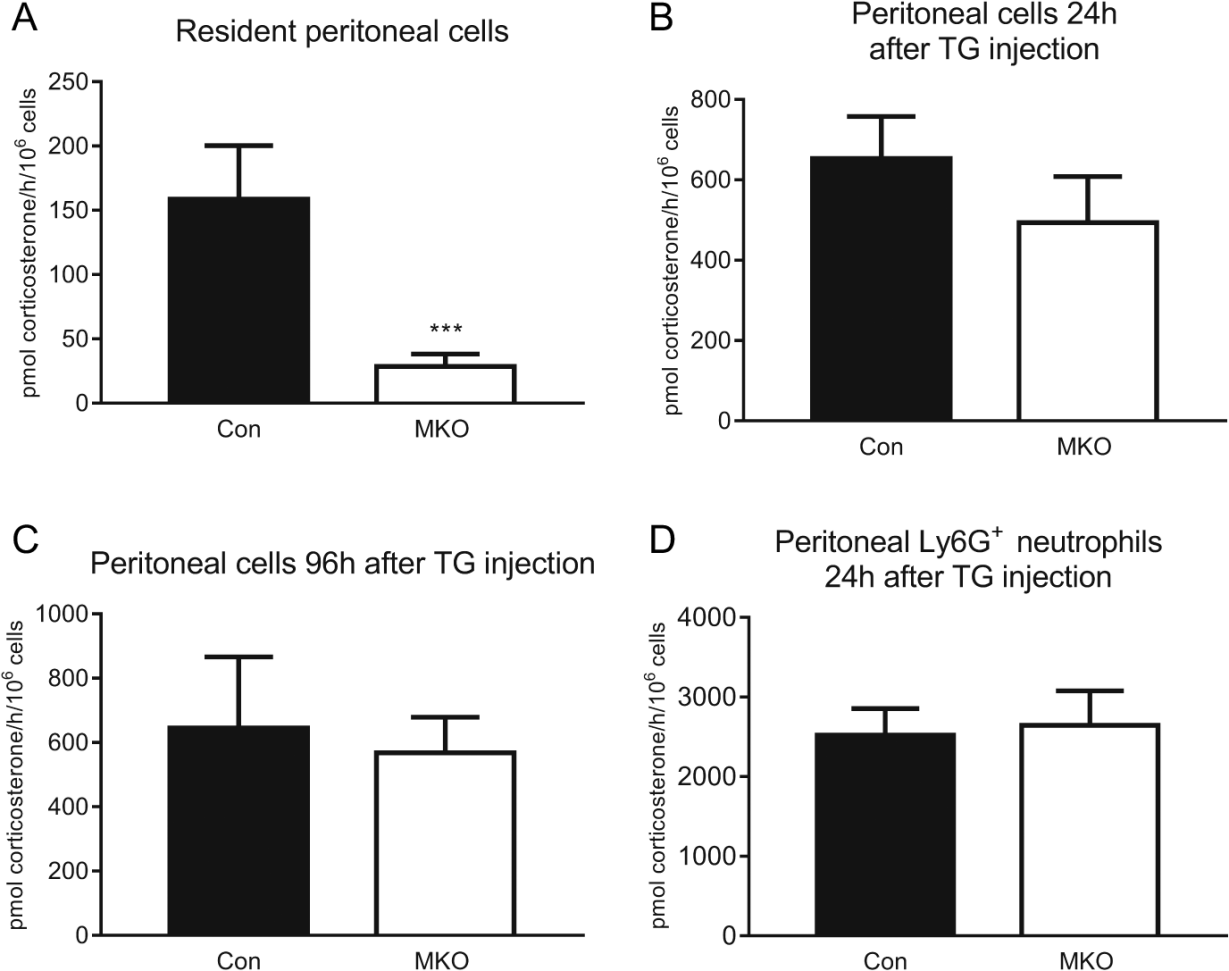
11β-HSD1 activity is markedly reduced in resident peritoneal macrophages from *Hsd11b1^MKO^* mice. Resident peritoneal cells, or cells elicited to the peritoneum by i.p. injection of 0.2 mL 10% thioglycollate (TG), were harvested from *Hsd11b1^MKO^* (MKO: white bars) and control *Hsd11b1*^f/f^** mice (Con: black bars). 11β-HSD1 activity in peritoneal cells was measured by conversion of [^3^H]-11-dehydrocorticosterone to corticosterone in resident peritoneal cells (A) and in cells elicited to the peritoneum 24 h (B) or 96 h (C) following thioglycollate injection. Activity was also measured in purified neutrophils (Ly6G^+^ cells) isolated from the peritoneum 24 h after thioglycollate injection (D). 11β-HSD1 activity is expressed as pmol corticosterone/h/10^5^ cells. Values are means ± s.e.m. and were analysed by unpaired *t*-test (*n* = 3–8, *P < 0.05).

To generate *Hsd11b1^MKO^* mice with disruption of 11β-HSD1 expression in myeloid phagocytes, *LysM-Cre* transgenic mice (Cramer *et al*. 2003) were crossed with mice in which exon 3 of the *Hsd11b1* gene is flanked by *LoxP* sites (*Hsd11b1^f/f^* mice) (recombination strategy shown in Supplementary Fig. 1). *LysM-Cre* transgenic mice are reported to efficiently delete *LoxP*-flanked target genes in granulocytes and mature macrophages, with lower efficiencies in other myeloid cells (Clausen *et al*. 1999). Measurement of 11β-HSD1 activity in myeloid cell populations from *Hsd11b1^MKO^* mice showed an 89% decrease in 11β-HSD1 activity in peritoneal resident cells, compared to *Hsd11b1^f/f^* littermate controls (Fig. 1A), consistent with the disruption of *Hsd11b1* in this predominantly macrophage population (Clausen *et al*. 1999). In contrast, there was no significant reduction in 11β-HSD1 activity in thioglycollate-elicited peritoneal cells from *Hsd11b1^MKO^* mice collected 24 h (monocytes/macrophages and neutrophils (Melnicoff *et al*. 1989, Coutinho *et al*. 2016)) or 96 h following thioglycollate injection (predominantly macrophages (Melnicoff *et al*. 1989, Zhang *et al*. 2008)) (Fig. 1B and C). Similarly, 11β-HSD1 activity was not significantly altered in purified neutrophils (Ly6G+ cells) from the peritoneum of thioglycollate-injected *Hsd11b1^MKO^* mice, compared to littermate controls (Fig. 1D). 11β-HSD1 activity was maintained in these myeloid cell populations despite the efficient reduction in *Hsd11b1* mRNA levels (Supplementary Fig. 2). Western blotting confirmed a marked reduction in 11β-HSD1 protein levels in the resident peritoneal cell population of *Hsd11b1^MKO^* mice, but only a modest reduction in peritoneal cells collected 24 h or 96 h following thioglycollate injection and in purified neutrophils (Supplementary Fig. 3). These data suggest that the protein half-life of 11β-HSD1 exceeds the half-life of the thioglycollate-elicited monocyte/macrophage and neutrophil populations, but that 11β-HSD1 activity is markedly reduced in the longer-lived resident macrophage population. Given this, we sought to test the role of macrophage 11β-HSD1 in models of inflammation in which the role of resident macrophages may be evident.

### Resolution of experimental arthritis is impaired in *Hsd11b1^MKO^* mice

**Figure 2:**
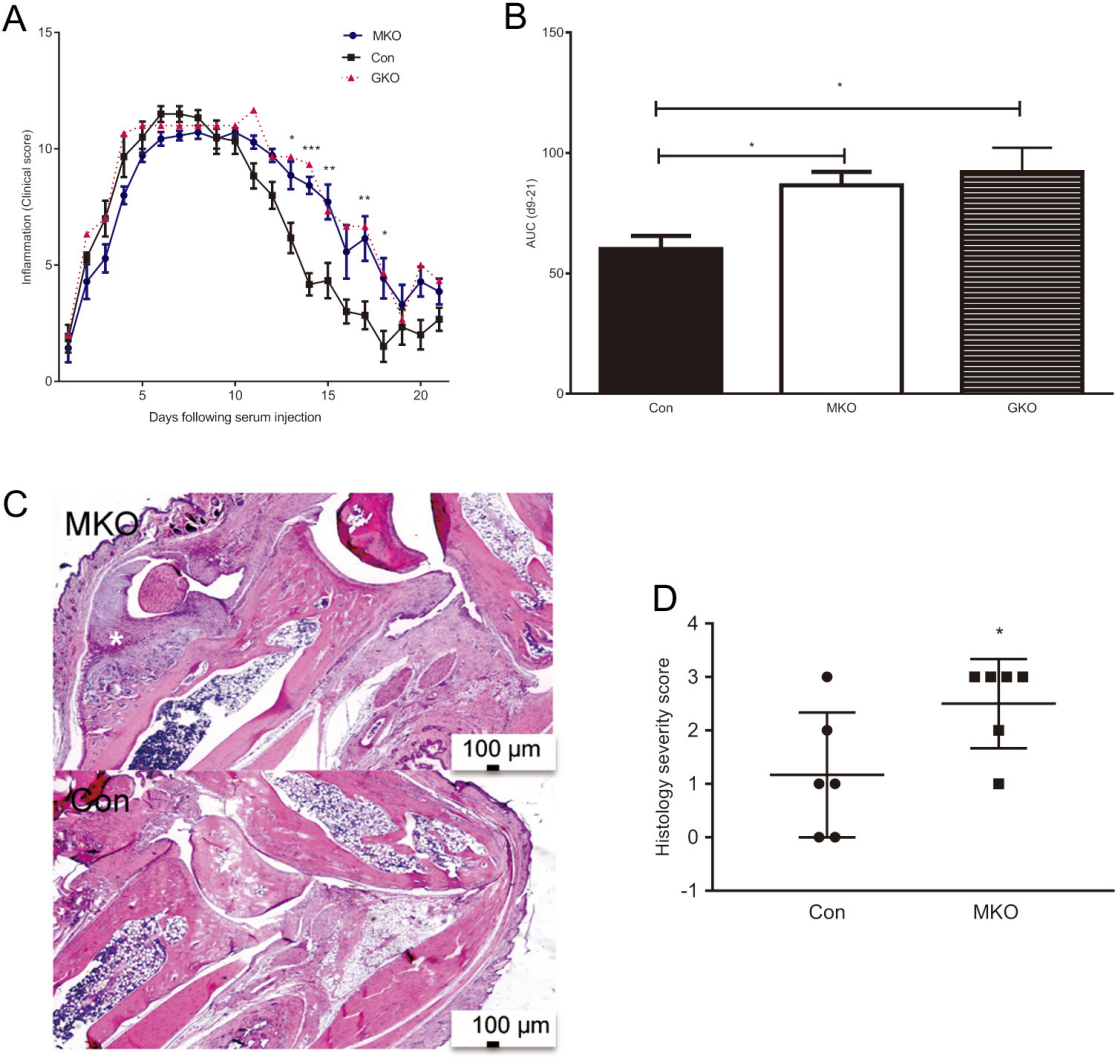
Resolution of K/BxN serum transfer-induced arthritis is impaired in *Hsd11b1^MKO^* mice. Arthritis was induced in *Hsd11b1^Del1/Del1^* (global knockout, GKO: triangular symbols, *n* = 3), *Hsd11b1^MKO^*(MKO: circular symbols/white bars, *n* = 6) and control *Hsd11b1^f/f^*mice (Con: square symbols/black bars, *n* = 7) by i.p. injection of 100 µL K/BxN serum at day 0. (A) Clinical scoring of joint inflammation over 21 days. Values are means ± s.e.m. for *Hsd11b1^MKO^* and control *Hsd11b1^f/f^* mice. Data were analysed by two-way repeated measurement analysis of variance (ANOVA) with Bonferroni’s multiple comparisons test; *P < 0.05, **P < 0.01, ***P < 0.001. Only the mean value is shown for *Hsd11b1^Del1/Del1^*mice. (B) The area under the curve (AUC) was calculated for the clinical score from days 9 to 21 (the resolution phase). Values are means ± s.e.m. Data were analysed by one-way ANOVA (*P* < 0.01) with *post-hoc* Dunnett’s multiple comparisons test with the *Hsd11b1^f/f^* group as control; *P < 0.05. (C) Representative sections of joints from *Hsd11b1^MKO^* and *Hsd11b1^f/f^* mice collected 21 days after injection and stained with haematoxylin and eosin showing tenosynovitis characterised by synovium hyperplasia, bone erosion and new bone formation. Scale bars, 100 µm. (D) Histopathological changes were quantified using a scoring index (see the ‘Materials and methods’ section for details) by an investigator blind to genotype. Values are means ± s.e.m. Data were analysed by Mann–Whitney test, *n* = 6, *P < 0.05.

Tissue-resident macrophages have been implicated in the development and resolution of inflammatory arthritis in mice induced by injection of K/BxN serum (Misharin *et al*. 2014). We therefore investigated whether macrophage 11β-HSD1 deficiency contributes to the more severe arthritis that develops in mice globally deficient in 11β-HSD1 following injection of arthritogenic K/BxN serum (Coutinho *et al*. 2012). *Hsd11b1^MKO^* mice did not replicate the earlier onset of arthritis seen previously with global 11β-HSD1 deficiency (Coutinho *et al*. 2012). However, resolution of arthritis was impaired in *Hsd11b1^MKO^* mice, compared to *Hsd11b1^f/f^* controls (Fig. 2A and B). The course of the resolution phase in *Hsd11b1^MKO^* mice was very similar to that of *Hsd11b1^Del1/Del1^* mice globally deficient in 11β-HSD1 (Fig. 2A and B). Histopathological examination of the joints showed bone erosion and marked fibroproliferation in the synovium (Fig. 2C). Quantification of histopathology using a scoring index showed more marked histopathological changes in *Hsd11b1^MKO^* mice, compared to littermate controls (Fig. 2D).

**Figure 3:**
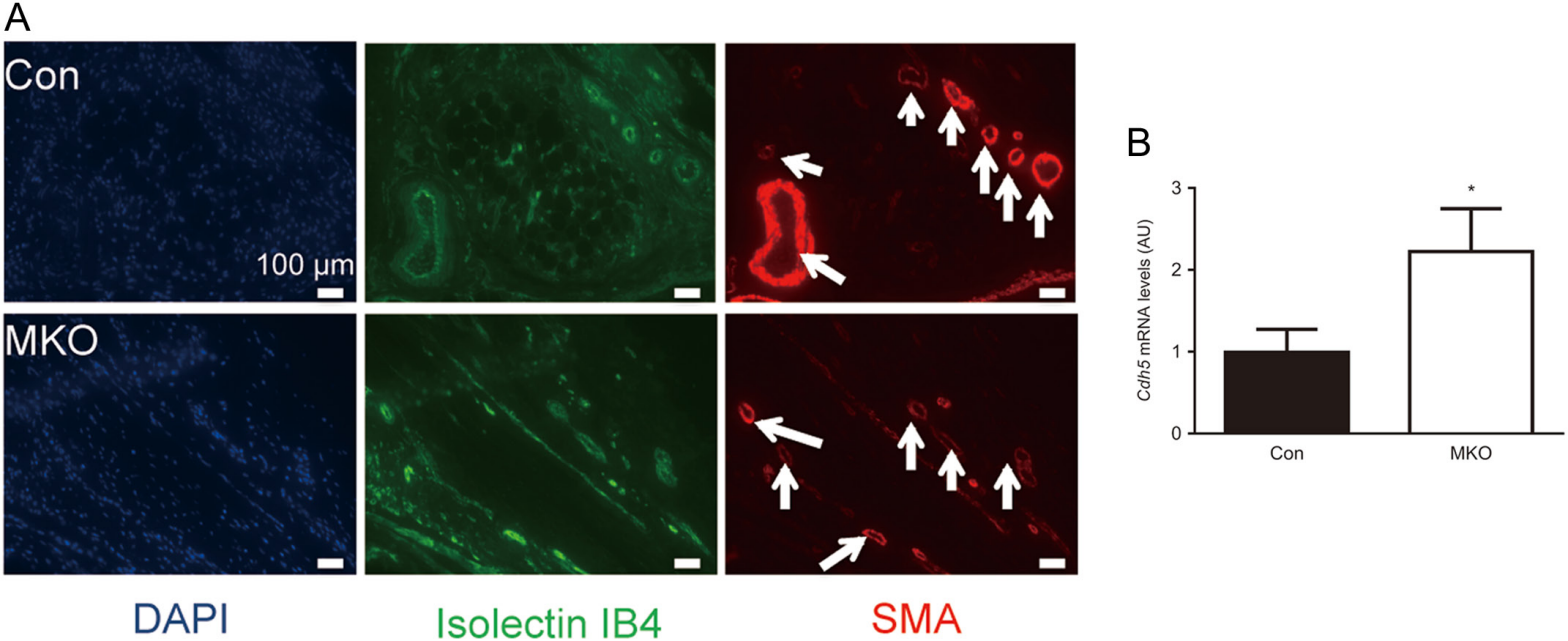
Higher *Cdh5* expression suggests greater angiogenesis in the joints of *Hsd11b1^MKO^* mice. Arthritis was induced by i.p. injection of 100 µL K/BxN serum, and the mice were killed 21 days later. (A) Immunofluorescent staining of blood vessels in the mesenchymal tissue of *Hsd11b1^MKO^* (MKO: top) and control mice (Con: bottom). From left to right: DAPI, isolectin B4, α-SMA. (B) RNA was extracted from hind joints and qPCR used to measure levels of *Cdh5* mRNA relative to *Hprt*, used as an internal standard. Values are in arbitrary units (AU) and are means ± s.e.m. Data from *Hsd11b1^MKO^* (white bar) and *Hsd11b1^f/f^* mice (black bar) were analysed by unpaired *t*-test, *n* = 5–7, *P < 0.05.

Rheumatoid arthritis is associated with neo-angiogenesis within the affected joint (Jackson *et al*. 1997, Koch 1998). Consistent with this, immunofluorescent staining of joints for the endothelial cell marker, isolectin IB4, and α-smooth muscle actin (α-SMA), a marker of perivascular mural cells, revealed a high degree of vascularisation of the soft tissues of joints of both *Hsd11b1^MKO^* and control mice following K/BxN serum transfer arthritis (Fig. 3A). However, qPCR measurement of *Cdh5* mRNA encoding the endothelial marker, vascular endothelial (VE)-cadherin, showed >2-fold higher levels of *Cdh5* mRNA in joints of *Hsd11b1^MKO^* mice, compared to littermate controls (Fig. 3B).

### Greater inflammatory angiogenesis in *Hsd11b1^MKO^* mice following subcutaneous sponge implantation

**Figure 4:**
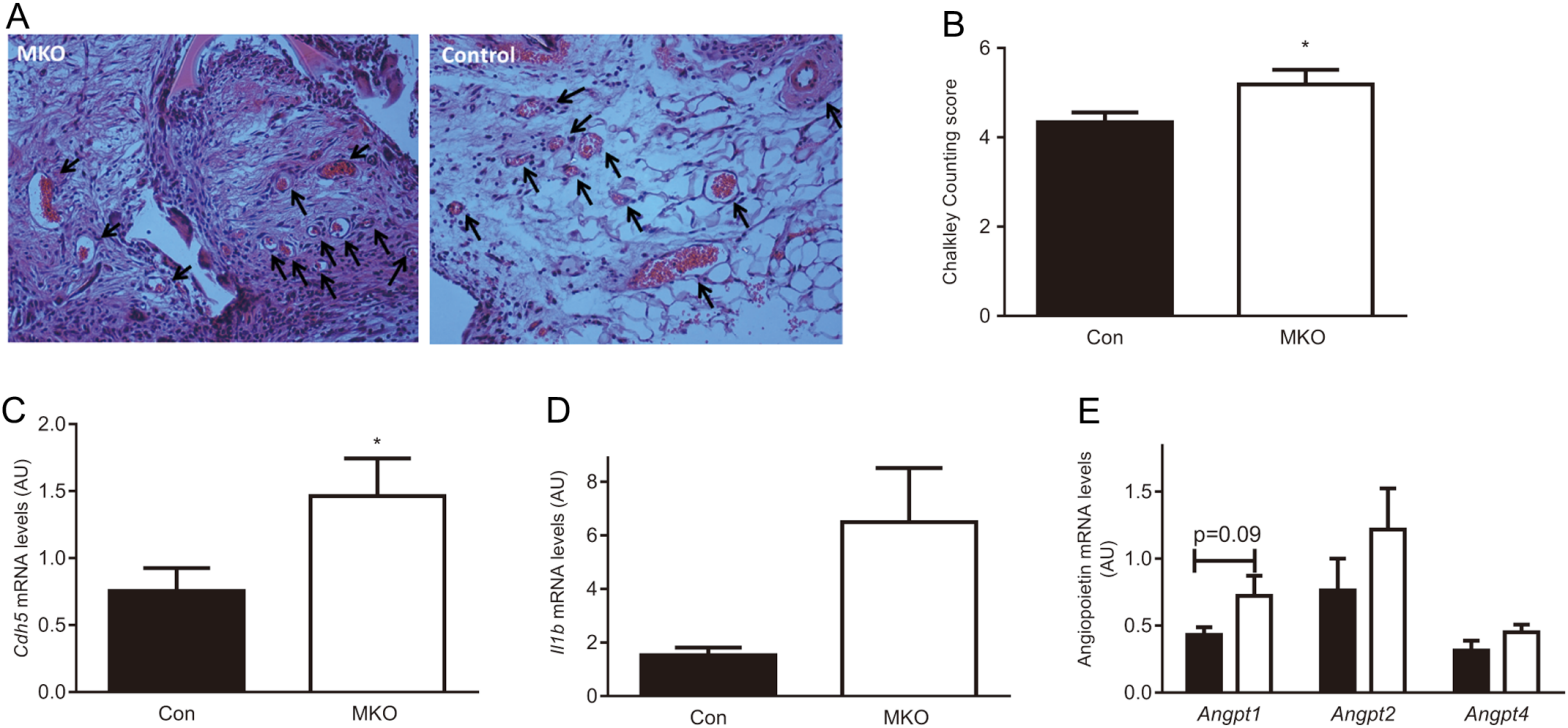
Compared to littermate controls, *Hsd11b1^MKO^* mice show greater angiogenesis in the subcutaneous sponge implantation assay. (A) Representative images (×125 magnification) of haematoxylin and eosin-stained sections of implanted sponges removed after 21 days, showing neovascularisation (blood vessels: arrows). (B) Quantification of blood vessel numbers by Chalkley counting. RNA was extracted from sponges removed 21 days after implantation, and qPCR was used to measure levels of: (C) *Cdh5* mRNA, (D) *Il1* mRNA, and (E) *Angiopoietin-1, -2* and *-4* mRNAs, relative to levels of *Hprt* mRNA, used as an internal standard (mRNA values in arbitrary units, AU). Values are means ± s.e.m. Data from *Hsd11b1^MKO^* (MKO: white bars) and *Hsd11b1^f/f^* mice (Con: black bars) were analysed by unpaired *t*-test (A, B, C, E) or unpaired *t*-test with Welch’s correction (D); *P < 0.05, *n* = 10–11.

Increased levels of *Cdh5* mRNA in joints of *Hsd11b1^MKO^* mice suggest an increase in neovascularisation of the joint during inflammatory arthritis. Mice with global 11β-HSD1-deficiency show greater inflammatory angiogenesis induced by subcutaneous sponge implantation (Small *et al*. 2005). To test whether macrophage 11β-HSD1 influences inflammatory angiogenesis, sponges were implanted subcutaneously in *Hsd11b1^MKO^* and littermate control mice, with angiogenesis assessed 21 days later (Figure 4A). Quantification of blood vessel density in sponges showed 20% greater vessel density in *Hsd11b1^MKO^* mice (Fig. 4A and B) associated with almost 2-fold higher levels of *Cdh5* mRNA (Fig. 4C). Levels of mRNA encoding IL-1β, a pro-angiogenic cytokine (Voronov *et al*. 2003), were >3-fold increased in sponges from *Hsd11b1^MKO^* mice (Fig. 4D) and there was also a trend for increased expression of mRNA encoding pro-angiogenic angiopoietins (Fig. 4E). Consistent with previous data from mice globally deficient in 11β-HSD1 (McSweeney *et al*. 2010), increased angiogenesis in *Hsd11b1^MKO^* mice was not associated with a change in *Vegfa* mRNA levels, nor were levels of mRNA encoding several other cytokines altered (Supplementary Fig. 4). These data support a role for macrophage 11β-HSD1 in controlling angiogenesis and thus promoting the resolution of inflammatory arthritis.

## Discussion

Our data in *Hsd11b1^MKO^* mice demonstrate that macrophages are a key cell type responsible for the pro-angiogenic phenotype conferred by 11β-HSD1 deficiency. This suggests that 11β-HSD1-mediated glucocorticoid regeneration within macrophages alters their phenotype to control an ongoing inflammatory response. In *Hsd11b1^MKO^* mice, this happens without the perturbation in the systemic ratio of active to inactive glucocorticoid levels that occurs with global 11β-HSD1 deficiency (Harris *et al*. 2001; Verma M, Kipari TMJ, Zhang Z, Man TY, Forster T, Homer NZM, Seckl JR, Holmes MC & Chapman KE, unpublished observations). Whether this is mediated by alterations in macrophage polarisation is currently unclear. However, *in vitro*, bone marrow-derived macrophages from *Hsd11b1^−/−^* mice behave similar to wild type (Gilmour *et al*. 2006, Zhang & Daynes 2007), suggesting the *in vivo* environment (and possibly substrate availability) is crucial to the physiological outcomes with 11β-HSD1 deficiency. This is suggested by evidence of an earlier macrophage polarisation to a pro-angiogenic ‘M2’ phenotype after myocardial infarction (McSweeney *et al*. 2010) and by higher expression of the M2 marker, SHIP1, in macrophages of *Hsd11b1^−/−^* mice than wild-type controls (Zhang & Daynes 2007).

Whilst resident peritoneal macrophages from *Hsd11b1^MKO^* mice showed the predicted reduction in 11β-HSD1 activity and *Hsd11b1* mRNA, our finding of near-normal 11β-HSD1 protein and activity in myeloid cells recruited to the peritoneum of thioglycollate-injected *Hsd11b1^MKO^* mice, despite a marked reduction in the encoding mRNA, was unexpected. We have previously reported a discrepancy between 11β-HSD1 protein/activity and levels of the encoding *Hsd11b1* mRNA in mouse neutrophils, with 11β-HSD1 protein present despite little encoding mRNA (Coutinho *et al*. 2016). Other studies have noted similar discrepancies in other cell types (Bujalska *et al*. 2005, Chinetti-Gbaguidi *et al*. 2012). This suggests that in most myeloid phagocytes 11β-HSD1 protein has a long half-life and persists without renewal from ongoing mRNA translation, at least when inflammatory conditions prevail. The reduction in 11β-HSD1 activity in resident peritoneal macrophages but not in thioglycollate-elicited macrophages may reflect the longer life span of the resident macrophage population compared to recruited bone marrow-derived macrophages (Davies *et al*. 2013). Alternatively, the *LysM-Cre* transgene may be more highly expressed in resident macrophages. Expression of *LysM/Lyz2*, encoding LYSM, is known to be heterogeneous in macrophage populations (Faust *et al*. 2000, Hume 2011). In peritoneal cells, expression of LYSM is reportedly highest in large peritoneal macrophages that are highly phagocytic (Lichanska *et al*. 1999) and correspond to the resident population (Cain *et al*. 2013). This implicates resident macrophages in the pro-angiogenic phenotype seen with 11β-HSD1 deficiency. Resident macrophages are important in tissue repair. They efficiently clear apoptotic cells and actively promote revascularisation (Uderhardt *et al*. 2012, Wang & Kubes 2016). Tissue-resident macrophages promote vessel anastomosis and thus vascular network complexity, acting downstream of vascular endothelial growth factor (VEGF)-α (Fantin *et al*. 2010). Our finding of unchanged expression of *Vegfa* in *Hsd11b1^MKO^* compared to control mice (also unchanged in global 11β-HSD1-deficient mice in a model of myocardial infarction (McSweeney *et al*. 2010)) is consistent with 11β-HSD1 acting in tissue-resident macrophages to restrain angiogenesis, downstream of VEGF-α. However, VEGF signalling is regulated at the post-translational level and further investigation is required to establish whether (or how) VEGF signalling is involved in the increased angiogenesis that occurs in *Hsd11b1^MKO^* mice. Our data also suggest resident macrophages are an important target of glucocorticoid action in the resolution of inflammation. However, it remains possible that, in addition to macrophages, other cell types contribute to the pro-angiogenic phenotype observed with global 11β-HSD1 deficiency.

Thus, *Hsd11b1^MKO^* mice support a role for 11β-HSD1 in restraining and shaping the healing response orchestrated by resident macrophages in ischaemic or injured tissue. These mice will be a useful tool with which to dissect the contribution of macrophages to beneficial (or otherwise) effects of 11β-HSD1 in other models of tissue repair and/or chronic inflammation.

## Supplementary Material

Supporting Figure 1

Supporting Figure 2

Supporting Figure 3

Supporting Figure 4

Supporting Table 1

## Declaration of interest

J R S holds patents on selective 11β-HSD1 inhibitors. None of the other authors has a conflict of interest.

## Funding

This work was supported by an MRC project grant (number 86642) and also by a Wellcome Trust programme grant (number 083184/Z/07/Z). Z Z was supported by a China Scholarships Council/University of Edinburgh Scholarship.
