# Supplementary material for: Macrophage 11β-HSD-1 deficiency promotes inflammatory angiogenesis
Source: J Endocrinol. 2017 Jul 4;234(3):291–9. doi: 10.1530/JOE-17-0223 (PMC5574305; doi:10.1530/JOE-17-0223)
Supplement: Supporting Figure 1 [file erc-234-291-s001.pdf]

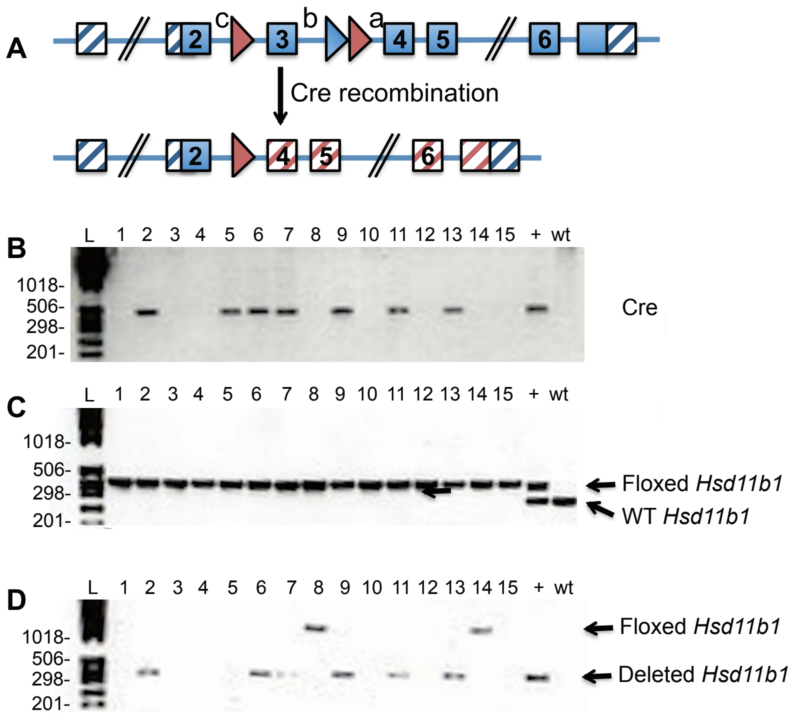

Supplementary Figure 1. Structure of the 'floxed' and MKO Hsd11b1 alleles. (A) Structure of the floxed *Hsd11b1* (*Hsd11b1*^{f}) allele: exons are indicated by boxes. Blue hatched boxes indicate untranslated regions, with solid boxes representing the 11β-HSD1 open reading frame. Lower case letters indicate positions of primers used for genotyping. FRT recombination was used to remove a selection cassette, leaving a single FRT element (blue triangle). In *Hsd11b1*^{MKO} mice, Cre-mediated recombination between *LoxP* sites (red triangles) removes exon 3 and generates a frameshift affecting the remaining exons (red hatched boxes). (B-D) show representative genotyping PCR reactions carried out on the same 15 mice, with controls containing wild-type (WT) or *Hsd11b1*$^{f/+}$*Cre*$^{+}$ (+) DNA. (B) Cre genotyping generates a 465bp product (arrow: mice 2, 5, 6, 7, 9, 11 and 13 are *Cre*$^{+}$). (C) PCR with primers a and b, flanking the 3' *LoxP* site, produces a 255bp product from the wild-type *Hsd11b1* allele (arrow: WT *Hsd11b1*) and a 385bp product from the 'floxed' *Hsd11b1*^{f} allele (arrow: Floxed *Hsd11b1*). As expected, none of the mice carry the WT allele. (D) Primers a and c produce a 363bp product from the *Hsd11b1*^{MKO} allele (arrow; Deleted *Hsd11b1*), present in all *Cre*$^{+}$ mice, and a 1066bp product from the floxed *Hsd11b1*^{f} allele (arrow; Floxed *Hsd11b1*), present in all *Cre*$^{-}$ mice.
