# Supplementary material for: Macrophage 11β-HSD-1 deficiency promotes inflammatory angiogenesis
Source: J Endocrinol. 2017 Jul 4;234(3):291–9. doi: 10.1530/JOE-17-0223 (PMC5574305; doi:10.1530/JOE-17-0223)
Supplement: Supporting Figure 2 [file erc-234-291-s002.pdf]

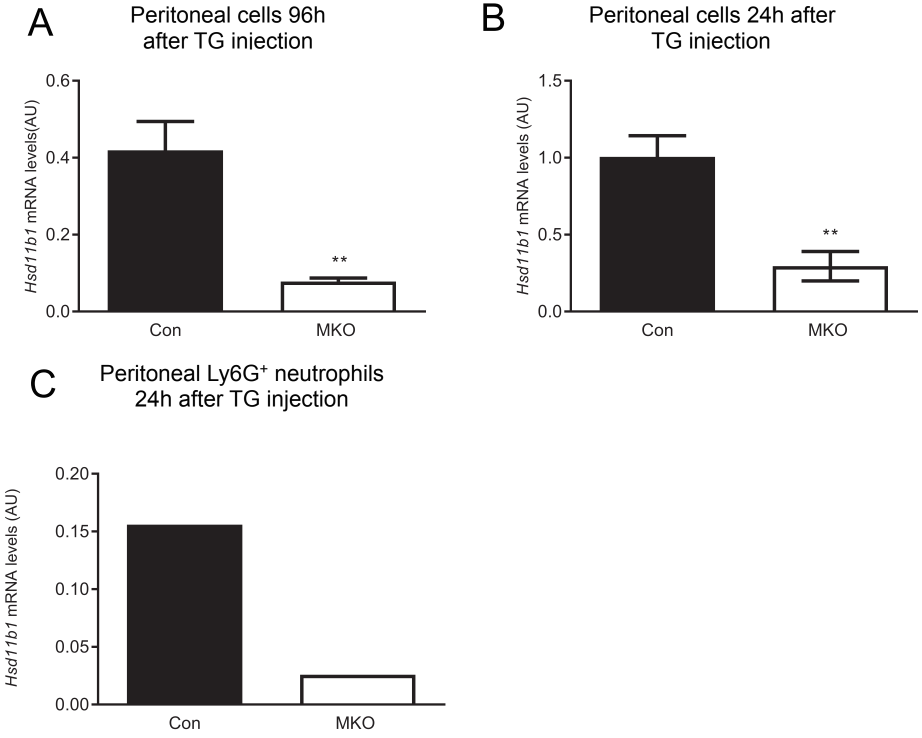

Supplementary Figure 2. *Hsd11b1* mRNA levels are reduced in *Hsd11b1*^{MKO} mice in cells elicited to the peritoneum by thioglycollate injection.
Hsd11b1^{MKO} (MKO: white bars) and control *Hsd11b1*$^{f/f}$ mice (Con: black bars) were injected (i.p.) with 0.2ml 10% thioglycollate (TG) and peritoneal cells harvested. *Hsd11b1* mRNA levels were measured by qPCR in (A) cells lavaged 96h after TG injection, (B) cells lavaged 24h after TG injection and (C) neutrophils, affinity purified using Ly6G antibody from cells harvested 24h after thioglycollate injection. *Hsd11b1* mRNA levels are expressed relative to levels of *Tbp* mRNA, used as internal control. (A, B) Data are means ± SEM and were analysed by unpaired t-test; ** $p<0.01$, n=4-6/group). For (C), cells were pooled from 3-4 mice and values are the mean of 2 (*Hsd11b1*^{MKO}) or 3 (*Hsd11b1*$^{f/f}$ mice) replicate samples.
