# Supplementary material for: Macrophage 11β-HSD-1 deficiency promotes inflammatory angiogenesis
Source: J Endocrinol. 2017 Jul 4;234(3):291–9. doi: 10.1530/JOE-17-0223 (PMC5574305; doi:10.1530/JOE-17-0223)
Supplement: Supporting Figure 3 [file erc-234-291-s003.pdf]

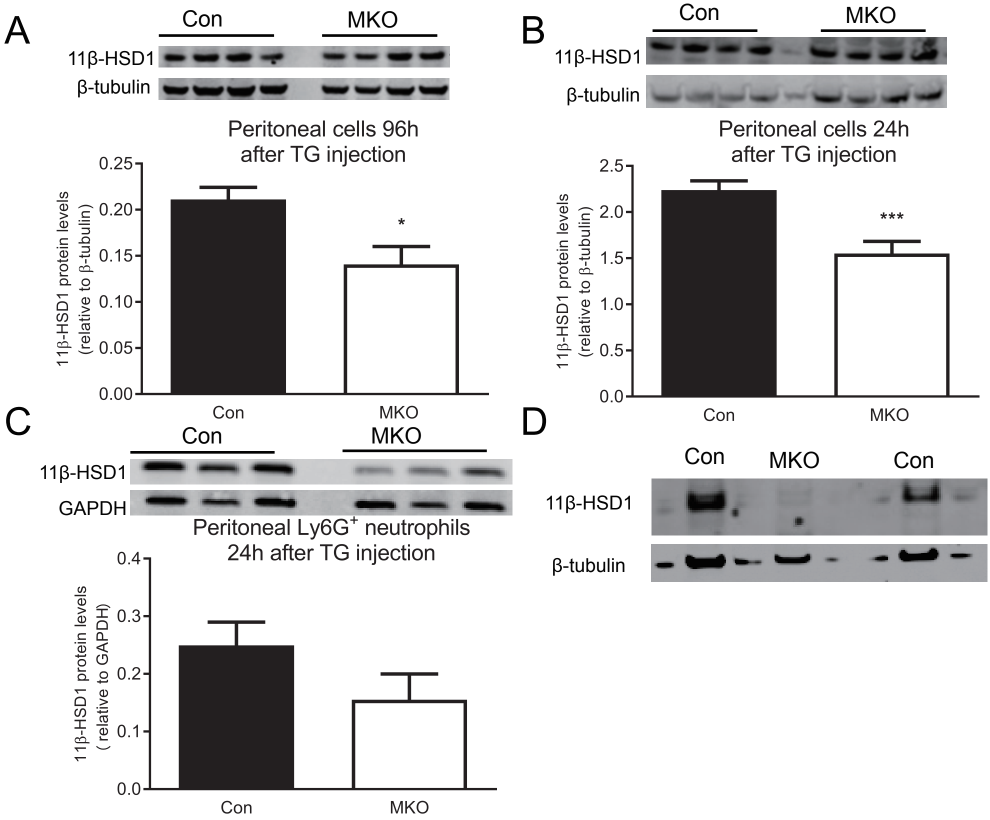

Supplementary Figure 3. A modest decrease in 11β-HSD1 protein levels in myeloid cells elicited to the peritoneum of *Hsd11b1^{MKO}* mice by thioglycollate.

Western blotting was used to measure 11β-HSD1 protein levels in myeloid cells from *Hsd11b1^{MKO}* (MKO: white bars) and control *Hsd11b1$^{f/f}$* mice (Con: black bars). Quantification of 11β-HSD1 protein levels in (A) Peritoneal macrophages lavaged 96h after thioglycollate injection, measured relative to levels of β-tubulin, (B) Total peritoneal cells lavaged 24h after thioglycollate injection, measured relative to levels of β-tubulin, (C) Ly6G-affinity purified neutrophils isolated from cells elicited to the peritoneum 24h after thioglycollate injection, measured relative to levels of GAPDH and (D) Resident peritoneal cells (1 mouse sample/lane; note – only 3 lanes were loaded; bands in other lanes are due to spill-over). Data are means ± SEM and were analysed by unpaired t-test; *p<0.05, ***p<0.001, n=4/group (A, B) or 3/group (C).
