# Supplementary material for: Macrophage 11β-HSD-1 deficiency promotes inflammatory angiogenesis
Source: J Endocrinol. 2017 Jul 4;234(3):291–9. doi: 10.1530/JOE-17-0223 (PMC5574305; doi:10.1530/JOE-17-0223)
Supplement: Supporting Figure 4 [file erc-234-291-s004.pdf]

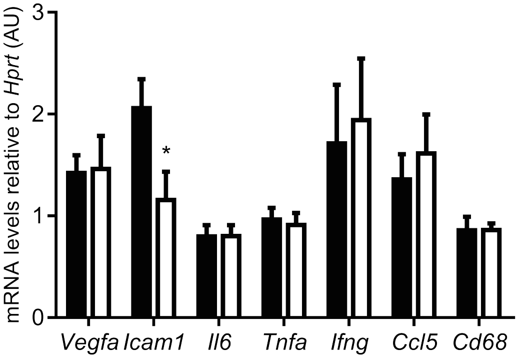

Supplementary Figure 4. Expression of inflammation-related genes is largely unchanged in sponges recovered from *Hsd11b1^{MKO}* mice, compared to littermate controls.
RNA was extracted from sponges removed 21 days after implantation and qPCR was used to measure levels of *Vegfa*, *Icam1*, *Il6*, *Tnfa*, *Ifng*,*Ccl5* and *Cd68* mRNA, relative to *Hprt* mRNA, used as an internal standard. Values are in arbitrary units (AU) and are means ± SEM.
Data from *Hsd11b1^{MKO}* (white bars) and *Hsd11b1$^{f/f}$* mice (black bars) were analysed by unpaired t test, n=6-11, *$p<0.05$.
