# Supplementary material for: Macrophage 11β-HSD-1 deficiency promotes inflammatory angiogenesis
Source: J Endocrinol. 2017 Jul 4;234(3):291–9. doi: 10.1530/JOE-17-0223 (PMC5574305; doi:10.1530/JOE-17-0223)
Supplement: Supporting Table 1 [file erc-234-291-t001.pdf]

SUPPLEMENTARY TABLE 1, qPCR primers and probes

Gene	Primers	UPL Probe
hprt	Forward: tcctcctcagaccgctttt<br>Reverse: cctggttcatcatcgctaatc	95
TBP	Forward: gggagaatcatggaccagaa<br>Reverse: gatgggaattccaggagtca	97
18s	Forward: ctcaacacgggaaacctcac<br>Reverse: cgctccaccaactaagaacg	77
Hsd11b1	Forward: tctacaaatgaagagttcagaccag<br>Reverse: gccccagtgacaatcacttt	1
Il1b	Forward: tgtaatgaaagacggcacacc<br>Reverse: tcttctttgggtattgcttgg	78
Il6	Forward: gctaccaaactggatataatcagga<br>Reverse: ccaggtagctatggtactccagaa	6
Tnfa	Forward: tcttctcattcctgcttgtgg<br>Reverse: ggtctgggccatagaactga	49
Icam1	Forward: cccacgctacctctgctc<br>Reverse: gatggatacctgagcatcacc	81
Ifnr	Forward: ggaggaactggcaaaaggat<br>Reverse: ttcaagacttcaaagagtctgagg	21
Vegfa	Forward: aaaaacgaaagcgcaagaaa<br>Reverse: tttctccgctctgaacaagg	1
Angpt1	Forward: ggaagatggaagcctggat<br>Reverse: accagagggattcccaaaac	12
Angpt2	Forward: cacactgaccttccccaact<br>Reverse: cccacgtccatgtcacagta	82
Angpt4	Forward: cgcctggtacggattgtag<br>Reverse: tggaacactggcttaggtgtc	89
Cdh5	Forward: tcatcaaacccacgaagtcc<br>Reverse: ggtctgtggcctcaatgtaga	42
Cd68	Forward: gacctacatcagagcccgagt<br>Reverse: cgccatgaatgtccactg	96
